# Implementation of LED Fluorescence Microscopy for Diagnosis of Pulmonary and HIV-Associated Tuberculosis in a Hospital Setting in Indonesia

**DOI:** 10.1371/journal.pone.0061727

**Published:** 2013-04-19

**Authors:** Lidya Chaidir, Ida Parwati, Jessi Annisa, Soni Muhsinin, Intan Meilana, Bachti Alisjahbana, Reinout van Crevel

**Affiliations:** 1 Health Research Unit, Faculty of Medicine, Universitas Padjadjaran/Hasan Sadikin Hospital, Bandung, Indonesia; 2 Department of Clinical Pathology, Faculty of Medicine, Universitas Padjadjaran/Hasan Sadikin Hospital, Bandung, Indonesia; 3 Department of Medicine, Radboud University Medical Centre, Nijmegen, The Netherlands; McGill University, Canada

## Abstract

**Background:**

Fluorescence microscopy (FM) has not been implemented widely in TB endemic settings and little evaluation has been done in HIV-infected patients. We evaluated diagnostic performance, time and costs of FM with light-emitting diodes technology (LED-FM), compared with conventional (Zieh-Neelsen) microscopy in a hospital in Indonesia which acts as referral centre for HIV-infected patients.

**Method:**

We included pulmonary tuberculosis suspects from the outpatient and HIV clinic. Direct and concentrated sputum smears were examined using LED-FM and ZN microscopy by two technicians who were blinded for the HIV-status and the result of the comparative test. Mean reading time per slide was recorded and cost of each slide was calculated. Mycobacteria culture served as the reference standard.

**Results:**

Among 404 tuberculosis suspects from the outpatient clinic and 256 from the HIV clinic, mycobacteria culture was positive in 12.6% and 27%, respectively. The optimal sensitivity of LED-FM was achieved by using a threshold of ≥2 AFB/length. LED-FM had a higher sensitivity (75.5% vs. 54.9%, P<0.01) but lower specificity (90.0% vs 96.6%, P<0.01) compared to ZN microscopy. HIV was associated with a lower sensitivity but similar specificity. The average reading time using LED-FM was significantly shorter (2.23±0.78 vs 5.82±1.60 minutes, P<0.01), while costs per slide were similar.

**Conclusion:**

High sensitivity of LED-FM combined with shorter reading time of sputum smear slides make this method a potential alternative to ZN microscopy. Additional data on specificity are needed for effective implementation of this technique in high burden TB laboratories.

## Introduction

Microscopic observation of *Mycobacterium tuberculosis* in sputum smears still remains the mainstay of tuberculosis (TB) diagnosis in developing countries despite its poor sensitivity [1] especially among people infected with HIV because of their lower bacterial burden [2], [3]. Fluorescence microscopy (FM) of auramine-stained smears has been studied as an alternative to conventional light microscopy with Ziehl Neelsen staining (ZN). In a systematic review published in 2006, FM showed a similar specificity and on average 10% higher sensitivity than ZN staining [4]. FM however is not widely implemented in many TB-endemic settings, one reason being the high costs of the microscope. This has changed with the advent of cheaper fluorescent microscopes with light-emitting diodes (LED) [5]. Studies evaluating the performance of LED-FM have shown that in addition to the higher sensitivity, it had qualitative, operational and cost advantages over both conventional FM and ZN. On the basis of these findings, the World Health Organization recommended in 2011 to replace conventional FM by LED-FM and phase in LED-FM as an alternative to ZN microscopy [6].

In spite of this recommendation, policy makers and laboratory staff in many settings seem reluctant to introduce LED-FM for TB diagnosis. This is also true for Indonesia which has the fifth highest TB case-load worldwide. Issues including quality control, acceptibility, and ease may hamper its introduction. In addition, accuracy data for LED-FM in HIV-infected patients are scarce, with only one published study to our knowledge [7]. Also, WHO advocates a low threshold for positivity of FM (≥1 acid-fast bacillus/smear) [8], but this low threshold may contribute to the lower specificity of LED-FM compared to conventional microscopy reported by several studies [9], [10], [11]. Finally, only limited studies have evaluated the possible effect of sputum processing on the preformance of LED-FM [7], [12]. In conclusion, additional data are needed to define the optimal technical conditions of LED-FM, and its performance under field conditions and among HIV-infected patients [13], [14], [15].

We therefore defined the optimal threshold for positivity of LED-FM, examined the role of sputum concentration, and compared the performance of LED-FM and conventional light microscopy in patients with and without HIV infection, in terms of diagnostic accuracy, time and running costs under field conditions in Indonesia. Mycobacterial culture on Ogawa solid medium was applied as the reference standard.

## Methods

### Ethics Statement

We used anonymized sputum samples collected as part of the study “The clinical features of pulmonary tuberculosis compared with pleural tuberculosis patients and the comparison of Auramin-stained method and Ziehl-Neelsen microscopy in both groups of patients” approved by the Ethical Committee of Hasan Sadikin Hospital/Faculty of Medicine of Universitas Padjadjaran, Bandung, Indonesia (no. 91/FKUP-RSHS/KEPK/Kep/EC/2008). In addition, sputum samples were collected as part of laboratory assessment under an ongoing cohort study among HIV-infected patients in Hasan Sadikin hospital. Written informed consent is obtained from all patients in this cohort and this study has been approved by the Ethical Committee of Hasan Sadikin Hospital/Faculty of Medicine of Universitas Padjadjaran, Bandung, Indonesia (no.114/FKUP-RSHS/KEPK/Kep/EC/2007).

### Setting, Patients and Study Design

This study was conducted at Dr. Hasan Sadikin Hospital, the referral hospital for West Java Province, Indonesia. We included two groups of patients with suspected pulmonary TB, defined by the presence of cough ≥2 week duration with or without chest X-ray (CXR) abnormalities. The first group (‘group 1’) consisted of 527 outpatients, not (yet) taking TB treatment and with a very low risk of being HIV-infected. HIV testing is not routinely done for all pulmonary TB suspects in Indonesia. So far, Indonesia has a concentrated HIV epidemic and two large cohort studies, one in our research setting, have shown an HIV-prevalence <2% among regular pulmonary TB patients [16], [17]. From this first group one sputum sample was used per patient. The second group (‘group 2’) consisted of 263 HIV-infected patients either from the outpatient clinic or the HIV clinic in the hospital who were screened for pulmonary TB. From this second group one to three consecutive sputum samples were collected per patient. All sputum samples were examined using ZN and LED-FM microscopy, and culture on Ogawa solid medium as a gold standard.

The study consisted of three parts. First, the optimal threshold (minimum number of AFB observed) of LED-FM in terms of sensitivity and specificity was established in a subgroup of patients from group 1. Then, the possible benefit of pretreatment of sputum in terms of sensitivity and specificity of microscopy was examined in a subgroup of specimens from group 1. Finally, accuracy, reading time and costs of LED-FM were compared with conventional light microscopy in both groups of patients.

### Smear Preparation, Microscopy and Culture

Direct smears were prepared in duplicate for staining by ZN and Auramin O. To examine the effect of sputum processing on the performance of both methods, smears of 102 specimens from group 1 were prepared in duplicate from sputum sediment after decontamination. Staining using ZN was carried out according to standard published procedure, and slides were examined with bright-field microscopy (Olympus CX21) at 1000×magnification. The microscopists in our laboratory have >10 years experience in ZN microscopy, and all participate and qualify in recommended EQA programs. ZN smears were read as part of routine laboratory work. Smears for FM were stained using auramin O. Briefly, smears were flooded with Auramin O for 10 minutes, destained with acid alcohol for 2 minutes, and then counterstained with potassium permanganate for one minute. With auramine O staining, mycobacteria appear as bright yellow fluorescent rods on a dark background. The slides were examined with CyScope (Partec, Germany) at 400×magnification. Auramin-stained smears were mostly read the same day they were prepared. A small proportion of stained smears could not be read the same day. They were stored at 4–8°C and all were read within 24 hours after staining. The presence or absence of AFB was reported using WHO/IUATLD guideline [18]. All smears were read by three micoscopists, all of whom are involved in routine diagnostic services. They all read similar proportions of both Auramin- and ZN-stained slides, without being aware of patient characteristics and results of comparitive tests.

Mycobacterial culture on Ogawa medium was used as the reference standard. Specimens were decontaminated by a standard N-acetyl-L-cystein (NaLC)-NaOH method and concentrated by centrifugation at 3000×g for 10 minutes. The resulting sediments were then resuspended in phosphate-buffered saline and directly inoculated onto Ogawa 3%. Cultures were considered positive when mycobacterial growth ≥1 colony forming unit (CFU) was observed within 8 weeks of incubation.

### Quality Control

We included positive (H37Rv strain) and negative (sterile distilled water) controls with each new batch of ZN and Auramin stain. Our lab also participates in EQA program for smear microscopy provided by the WHO. For mycobacterial culture, we monitor the contamination rate on a monthly basis, as a quality control for the decontamination process. All positive cultures are confirmed for the presence of AFB by using ZN microscopy. *M. tuberculosis* identification in our laboratory is done by spoligotyping. Genotyping of >800 cultured isolated in our laboratory has only revealed a very low number of non-tuberculous mycobacteria (<3%) [19].

### Data Analysis and Statistics

Test accuracy results were calculated as sensitivity and specificity along with 95% confidence intervals, using mycobacterial culture as a reference standard. For many subjects in the second group (‘HIV-infected’) more than one sputum sample was available, but analysis was done per patient, not per individual sample. Test characteristics (sensitivity and specificity) of LED-FM and conventional light microscopy were compared using the Chi-square test, with P-values <0.05 considered statistically significant. Receiver operating characteristic (ROC) analysis was performed using the non-parametric method, and values were expressed as AUC with 95% confidence intervals (95% CI). All analysis were performed using SPSS 13.

## Results

### Patients and Samples

A total of 527 suspects were recruited into group 1. Of these, cultures were contaminated in 19 patients and the results from either ZN or LED-FM results were lacking in 2 patients, leaving 506 patients for further analysis. Of these patients, 52% were male, the median age was 45 years (IQR 31–58), and mycobacterial culture was positive in 64 (12,6%) patients. In group 2 (HIV-infected patients), a total of 263 suspects were included. Of these, cultures were contaminated in 3 patients, and the smear results were unavailable in 4 patients, leaving 256 patients for analysis. Of these patients, 71% were male, the median age was 29 years (IQR 27–33), and the median CD4 count 57 cells/ml (IQR 13–238 cells/ml, available for 92% of patients). Mycobacterial culture was found positive in 69 (27%) patients in group 2.

### Comparison of LED-FM and ZN Using Different Cut-offs for Microscopy

**Figure 1:**
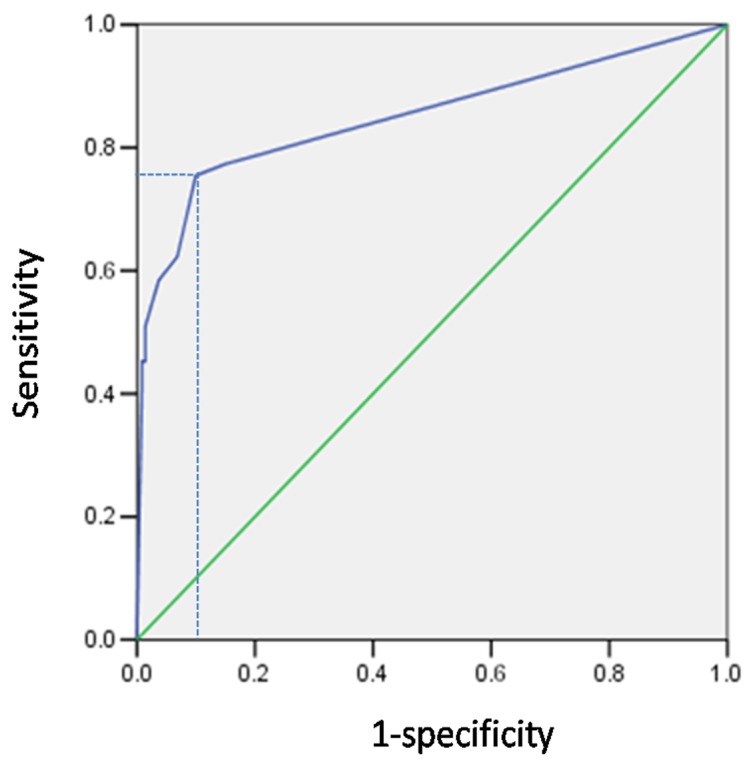
ROC curve for LED-FM. The optimal threshold for sensitivity and specificity of LED-FM was assessed using ROC analysis. Maximum area under the curve (AUC 0.85, 95% CI 0.78–0.92) was obtained at threshold of ≥2 AFB/length (sensitivity 75,5%, specificity 90,0%,). ROC = receiver operating characteristic, LED-FM =  Light emitting diode-fluorescence microscopy, AFB = Acid Fast Bacilli, CI = confidence interval.

Using samples from group 1, we first determined the most appropriate number of AFB to be used as a cut-off for LED-FM. Using the current WHO/IUTLD cut-off of a single AFB (≥1 AFB/length) in at least one single sputum smear, LED-FM had a much higher positivity rate compared to ZN (23% vs 10%, P<0.01). By grading the positive smear, it appeared that this was mostly due to a higher frequency of “scanty” (1–19 AFB/length) smears (**Table 1**). However, as shown in this table the specificity of scanty smears was much lower. By using the WHO threshold of ≥1 AFB/length, LED-FM had a higher sensitivity (77,4% vs 52,8%, P<0.01) but a lower specificity compared to conventional microscopy (84,9% vs 96,6%, P<0.01). The impact of scanty results of LED-FM on test performance was explored using ROC analysis **(** **Figure 1** **)**. The maximum area under the curve (AUC) was obtained at threshold of ≥2 AFB/length (sensitivity 75,5%, specificity 90,0%, AUC 0.85, 95% CI 0,78–0,92). At this threshold, the sensitivity of LED-FM was significantly higher compared to ZN at a threshold ≥1 AFB/length (75.5% vs 52.8%, P<0.01) but the specificity remained lower (90% vs 96.6%, P<0.01).

**Table 1:** Proportion of positive smears confirmed with culture.

Positive smear grading scale*	LED-FM		ZN	
	n = 94	positive by culture	n = 40	positive by culture
Scanty	69	19 (27,5%)	10	6 (60%)
+1	6	5 (83,3%)	11	6 (54,5%)
+2	4	3 (75%)	3	2 (66,7%)
+3	15	14 (93,3%)	16	14 (87,5%)

* smear results were reported by using the grading scales for ZN at 1000×magnification and FM at 400×magnification from WHO/IUATLD [18].

### Possible Benefit of Sputum Pretreatment

Smears of 102 specimens from patients in group 1 were prepared after sputum decontamination to examine the possible benefit of pretreatment of sputum in the performance of microscopic examination. **Table 2** presents results of the LED-FM and ZN performance using direct compared to concentrated smears. The sensitivity and specificity of both methods were better in concentrated versus direct smears, but both in concentrated and unconcentrated samples LED-FM had a somewhat lower specificity compared to ZN (**Table 2**).

**Table 2:** Accuracy of LED-FM and ZN method in direct and decontaminated smears.

	direct smears (n = 404)	decontaminated smears (n = 102)
LED-FM		
Sensitivity (95% CI)	75,5 (71,3–79,7)	81,8 (74,3–89,3)
Specificity (95% CI)	90,0 (87,1–93,0)	91,2 (85,7–96,7)
ZN		
Sensitivity (95% CI)	52,8 (47,9–57,7)	72,7 (64,0–81,3)
Specificity (95% CI)	96,6 (94,8–98,4)	97,8 (95,0–100)

LED-FM = Light emitting diode-fluorescence microscopy, ZN = Ziehl-Neelsen, CI = confidence interval.

### Diagnostic Performance in HIV-infected Individuals

The performance of sputum microscopy may be different in HIV-infected patients. In a group of patients with advanced HIV infection, the sensitivity of LED-FM seemed slightly higher compared to ZN, although this difference was not statistically significant (65.2% vs 58.0%, P = 0.09), while the specificity was lower (90,4% vs 96,3%, P<0.01). Compared with the first group of patients, most of whom were probably HIV-negative, LED-FM had a lower sensitivity among HIV-infected patients (65.2% vs 75.5%, P<0.01), but similar specificity **(** **Table 3** **)**.

**Table 3:** Accuracy of LED-FM and ZN in two study groups.

	LED-FM	ZN
Study group 1: Unknown HIV status (n = 404)		
Sensitivity (95% CI)	75,5 (71,3–79,7)	52,8 (47,9–57,7)
Specificity (95% CI)	90,0 (87,1–93,0)	96,6 (94,8–98,4)
PPV (95% CI)	53,3 (48,4–58,2)	70,0 (65,5–74,5)
NPV (95% CI)	96,0 (94,8–98,4)	93,1 (90,6–95,6)
Study group 2: HIV-positive (n = 256)		
Sensitivity (95% CI)	65,2 (59,4–71,0)	58,0 (52,0–64,0)
Specificity (95% CI)	90,4 (86,8–94,0)	96,3 (94,0–98,6)
PPV (95% CI)	71,4 (65,9–77,0)	85,1 (80,7–89,5)
NPV (95% CI)	87,6 (83,6–91,6)	86,1 (81,9–90,3)

LED-FM = Light emitting diode-fluorescence microscopy, ZN = Ziehl-Neelsen, PPV = positive predictive value, NPV = negative predictive value, CI = confidence interval.

### Reading Time and Cost

Using data from 400 direct smears from group 1, the average reading time was 2 minutes and 14 seconds (2′14′′, SD: 47′′) for LED-FM compared with 5′82′′ (SD: 1′60′′) for ZN microscopy (P<0.01). The average running cost per slide read was similar for LED-FM compared with ZN ($1.7 vs $1.6).

## Discussion

In many resource-limited countries, diagnosis of TB is based on microscopy of ZN-stained smears. However, fluorescence microscopy of Auramin-stained smears may be more sensitive and more quick. LED microscopes have replaced conventional halogen fluorescent microscopes and have brought fluorescent microscopy and Auramin staining into the reach of resource-limited countries [20]. In 2010, the WHO recommended that LED microscopy using Auramin staining be phased in as an alternative for conventional ZN microscopy in both high- and low-volume laboratories [6]. However additional data in high TB burden settings are needed to define the optimal technical conditions and its performance, especially among HIV infected patients.

We evaluated the performance of LED-FM in a hospital setting in Indonesia. LED-FM was more sensitive but less specific than ZN for the diagnosis of pulmonary TB both in unselected patients with TB and in HIV-infected patients. The diagnostic accuracy of LED-FM was better if a threshold of ≥2 AFB/length of the smear was used instead of the WHO threshold of ≥1 AFB/length. When sputum was concentrated before smear preparation, sensitivity and specificity of LED-FM increased. In HIV-infected patients, most of whom had advanced disease, LED-FM showed a slightly higher sensitivity and lower specificity compared with conventional ZN-microscopy. Finally, reading time of LED-FM was less than half from ZN-microscopy, while running costs were similar.

In line with previous studies we found that LED-FM had a higher sensitivity, but a lower specificity compared to ZN-microscopy [11], [21]. By using the new definition of a positive smear (≥1 AFB/length) from WHO [8], LED-FM in our study showed a relatively low specificity, resulting in a significant number of false-positive results based on ‘scanty smears’. Using ROC analysis, ≥2 AFB/length appeared to be the optimal threshold for LED-FM.

Although the number of studies is limited, fluorescence microscopy has shown promising results for diagnosing TB in HIV-infected individuals. Two studies [22], [23] included in the systematic review [4] reported increased sensitivity of conventional FM. In a study from Kenya, FM was twice as sensitive as ZN for HIV-infected cases using culture as a gold standard [22]. A second study in India showed an incremental yield of 26% for FM over ZN staining among 200 patients with clinically and radiologically diagnosed TB, including 15% HIV-positive patients [23]. These studies were performed with conventional FM.

In our study among 256 HIV-infected patients, by using the new cut-off (≥2 AFB/length) LED-FM had a somewhat higher sensitivity (65.2% vs 58%), albeit lower specificity compared to ZN-microscopy (90.4% vs 96.3%). This is in line with one study among 426 patients from Uganda, 85% of whom were HIV-infected, published after Steingart’s systematic review [9]. This study showed that conventional FM was more sensitive than ZN (49% vs 38%), but less specific (81% vs 96%) due to many scanty FM results. In that study the optimal performance of FM was seen when cut-off >4 AFB/100 fields used to define positive smears [9]. Whitelaw et al compared the accuracy of LED-FM with ZN in 345 patients, including 88 who were HIV-infected patients [7]. Similar to our study LED-FM performed better than ZN staining when using unprocessed samples, with a lower sensitivity of LED-FM in HIV-infected patients compared to HIV-uninfected patients. To our knowledge our study is the second to evaluate the performance of LED-FM in HIV-infected patients.

The lower specificity of LED-FM compared with ZN in our study may have several explanations. First, as a gold standard we used solid culture, which may be false-negative, especially among HIV-infected patients with paucibacillary TB. The probability of obtaining a positive culture is related to the number of AFB in the specimen, with only about 50% of cultures of specimens with 1–2 AFB per-100 fields being identified as positive, increasing to 80% and 96,7% for specimens with scanty (1–9 AFB/100 fields) and “positive” AFB grades, respectively [18]. This suggests that at least some LED-FM positive, culture-negative patients may have incorrectly been classified as false-positive LED-FM. Of course, false positive LED-FM results may also have been due to technical errors and lack of experience or training of technicians. Technicians in this study were experienced, but the technique and LED-microscope had not been used prior to the study.

Most microscopic examinations for pulmonary TB are performed directly on smeared and stained preparations of unprocessed sputum [3]. Various methods of concentrating sputum based on centrifugation have been shown to increase diagnostic yield when used prior to microscopy [24]. Our results showed that decontamination and concentration of sputum may improve the diagnostic accuracy of both ZN-microscopy and LED-FM. This is a feasible option if culture is performed routinely for all samples received in a laboratory. However, this is not the case in our hospital, like in many other settings in Indonesia and elsewhere. As such, it seems unsuitable to be implemented in high-burden laboratories.

In addition to higher sensitivity, shorter reading time and the simplicity of the Auramin-O staining method also indicate the potential benefit of making LED-FM more widely implemented in settings with a high burden of TB. However, the appropiate and feasible external quality assurance (EQA) procedures are needed for TB laboratories planning to implement LED-FM because the fluorochrome-based stained fade over time [25].

WHO has recently endorsed the implementation of GeneXpert MTB/RIF assay for national TB programmes in developing countries as the first initial screening of TB in HIV [26]. To our knowledge, there is only one study that comprehensively compared the implementation of LED-FM with GeneXpert MTB/RIF assay. The results showed that the Xpert MTB/RIF assay outperformed LED microscopy in all type of specimens, and that all sputum smears reported as “scanty AFB” had a positive Xpert result. However, it is unlikely in the short term, that Xpert can be implemented widely to replace smear microscopy as the initial diagnostic test in Indonesia like in many other low-resource or high-volume settings. LED fluorescent microscopy may therefore be used as a reasonable and cost-effective alternative [27].

Our study has several limitations. First, our microscopist had experience with ZN microscopy and never used fluorochrome-based technique before the study was conducted, but we observed the increase of technical skill after training until they were confident to start this study. Second, we used solid culture as the reference standard, knowing that solid culture may be false-negative, especially in HIV-infected patients with paucibacillary disease. Specificity of LED-FM might in fact have been higher if liquid culture was used as a reference standard.

In conclusion, our study showed that LED-FM is more sensitive and more quick than conventional ZN-microscopy for TB diagnosis, but that a higher threshold for positivity than the current WHO-threshold may improve its specificity. This makes a suitable method and reasonable alternative to more sensitive but much more expensive molecular tests, especially in low-resource settings. However, implementation of LED-FM must be followed by adequate training, standardized protocols for staining and smear examination, and sustainable EQA systems.
